# A Head-to-Head Meta-Analysis of 35,052 Smooth vs Textured Implants in Breast Reconstruction and Augmentation

**DOI:** 10.1093/asjof/ojag032

**Published:** 2026-02-12

**Authors:** Yousef Tanas, Shadi Tanas, Julie Tanas, Liam Cato, Philong Nguyen, Joshua Wang, Hossam Ghorab, Sarya Swed, Aldona Spiegel

## Abstract

**Background:**

Whether texturing confers clinical advantages over smooth breast implants remains questionable, especially in the wake of breast implant–associated anaplastic large-cell lymphoma concerns with textured implants.

**Objectives:**

The aim of this study was to compare complications, implant-specific events, and patient-reported outcomes between smooth and textured implants across augmentation and reconstruction.

**Methods:**

Following PRISMA 2020, the authors synthesized head-to-head comparative studies through January 15, 2025. Random-effects models estimated risk ratios (RRs) for dichotomous outcomes and mean differences for BREAST-Q domains; heterogeneity (*I*^2^) guided prespecified subgroup and sensitivity analyses (eg, implant plane and exclusion of overlapping or historical cohorts). Review Manager v5.4 was used for statistical analysis.

**Results:**

Thirty-three studies comprising 35,052 implants met inclusion criteria. In the initial pooled analysis, smooth implants showed higher capsular contracture (RR = 1.69, 95% CI, 1.36-2.11, *P* < .00001; with significant heterogeneity, *I*^2^ = 79%, *P* < .00001); nonetheless, after stratifying by plane (subpectoral and prepectoral) and conducting sensitivity analyses that excluded overlapping/historical cohorts, the difference was no longer statistically significant (RR = 1.13, 95% CI, 0.82-1.56, *P* = .46) with no subgroup differences (*I*^2^ = 0%, *P* = .92). Infection was lower with smooth implants (RR = 0.51, 95% CI, 0.30-0.89, *P* = .02). No differences were detected for seroma, hematoma, rippling, rupture, malposition/rotation, explantation, or BREAST-Q domains.

**Conclusions:**

In contemporary, plane-matched comparisons with appropriate sensitivity analyses, smooth implants did not demonstrate a higher capsular contracture risk and were associated with lower infection risk compared with textured implants. Other complications and patient-reported outcomes were similar among both groups.

**Level of Evidence: 3 (Therapeutic):**

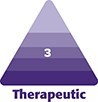

Silicone breast implants are a cornerstone of aesthetic and reconstructive breast surgery, with millions of devices implanted globally over the past 3 decades. Although long-term outcomes are generally favorable, capsular contracture remains the most common complication requiring reoperation, and ongoing debate surrounds the influence of implant surface texture on this risk. Early laboratory and clinical evidence suggested that textured implants might reduce the incidence of Baker grade III-IV contracture by disrupting periprosthetic capsule organization and limiting implant mobility.^1^ These findings led to the widespread adoption of macro-textured shells in both augmentation and reconstruction.^2-7^

However, subsequent clinical experience has yielded mixed results. Although some large registries (such as the Sientra core study) demonstrated significantly lower contracture rates with textured devices over 5- to 10-year follow-up, other series have shown no statistically significant difference in capsular behavior when different surgical techniques (eg, subpectoral or prepectoral with acellular dermal matrix [ADM]) and antibiotic irrigation protocols are used.^5--11^ Compounding the controversy, textured devices (particularly those with high surface area and aggressive macrotexturing) have been implicated in the development of breast implant–associated anaplastic large-cell lymphoma (BIA-ALCL), prompting product withdrawals and regulatory restrictions in several countries.^12-15^

Because textured devices fall out of favor in the United States and many surgeons transition to smooth implants, there is a need to clarify whether this shift compromises patient safety or aesthetic outcomes. In particular, it is unclear whether the purported protective effect of texturing on capsular contracture persists in contemporary cohorts managed with no-touch technique, dual plane or prepectoral placement, ADM, and other modern refinements. Previous meta-analyses have pooled heterogeneous study designs, device generations, and follow-up intervals, limiting their generalizability to today's clinical practice.

The objective of this study was to conduct an updated, comprehensive meta-analysis comparing smooth vs textured breast implants in terms of capsular contracture, surgical complications, implant-specific events, and patient-reported outcomes. By synthesizing head-to-head data across both augmentation and reconstruction cohorts, this review aims to clarify whether implant surface independently predicts adverse outcomes—and whether smooth implants offer an equivalent, superior, or inferior risk profile in the post-BIA-ALCL era.

## METHODS

This systematic review and meta-analysis was prospectively registered on PROSPERO (CRD420251012203) and conducted in accordance with the PRISMA-2020 checklist (Supplemental Figure 1).

### Search Strategy

A comprehensive search of PubMed, Scopus, and Web of Science was executed from database inception to January 15, 2025. The Boolean string combined synonyms for breast implants (“breast implant*,” “augmentation,” and “reconstruction”) with surface descriptors (“smooth,” “textured,” “macro-textured,” and “micro-textured”). No language, geographic, or publication-status limits were applied. The reference lists of eligible articles and previous reviews were hand-searched to capture additional studies.

### Eligibility Criteria

Comparative human studies (randomized, prospective, or retrospective cohort/case–control designs) that directly compared smooth with textured silicone implants in cosmetic augmentation or postmastectomy reconstruction and reported at least one of the following were included:

- capsular contracture (Baker grade III-IV);
- other surgical complications (seroma, hematoma, infection, explantation, skin flap, or implant loss);
- device-specific events (rupture, malposition/rotation, and rippling); or
- BREAST-Q patient-reported outcomes.

Animal studies, single-arm series, case reports, reviews, editorials, conference abstracts lacking extractable data, and studies with irretrievable duplicate cohorts were excluded. Studies comparing tissue expanders only were also excluded.

### Study Selection and Data Extraction

Two reviewers (S.T. and J.T.) screened titles/abstracts, then full texts, independently. Discrepancies were resolved by a third reviewer (Y.T.). Using a piloted form, the same 2 reviewers independently extracted study characteristics, baseline demographics, implant plane, ADM use, follow-up, and all predefined outcomes.

### Statistical Analysis

Pooled risk ratios (RRs) for dichotomous outcomes and inverse-variance weighted mean differences for continuous BREAST-Q domains were calculated using Mantel–Haenszel random-effects models in Review Manager v5.4. Heterogeneity was quantified with the χ^2^ test and *I*^2^ statistic; *I*^2^ > 50% prompted exploration by subgroup (implant plane: subpectoral, prepectoral, and mixed) and sensitivity analyses that sequentially excluded historical industry registries, overlapping interim reports, and outlier studies. Leave-one-out analyses judged result robustness. Potential small-study effects were inspected with funnel plots when ≥10 studies informed an outcome. Statistical significance was set at *P* < .05 (2-tailed).

We prespecified a sensitivity analysis excluding studies published before 2007 to reflect modern device generations, contemporary surgical techniques (eg, dual plane and prepectoral with ADM), and updated reporting standards. We also excluded overlapping publications to avoid duplication.

### Risk of Bias

Two reviewers qualitatively appraised nonrandomized comparative studies using the ROBINS-I domains (confounding, selection, classification of interventions, deviations from intended interventions, missing data, outcome measurement, and selection of reported results). In keeping with ROBINS-I guidance, we did not compute numeric scores or sums across domains; instead, we reported a concise domain-level narrative and used these judgments to interpret pooled estimates and plan sensitivity analyses.

### Protocol Deviations

All methodological decisions (eg, exclusion of overlapping Sientra core publications) were predefined in the PROSPERO protocol; no unplanned deviations occurred.

## RESULTS

### Summary of Studies

**Figure 1.**
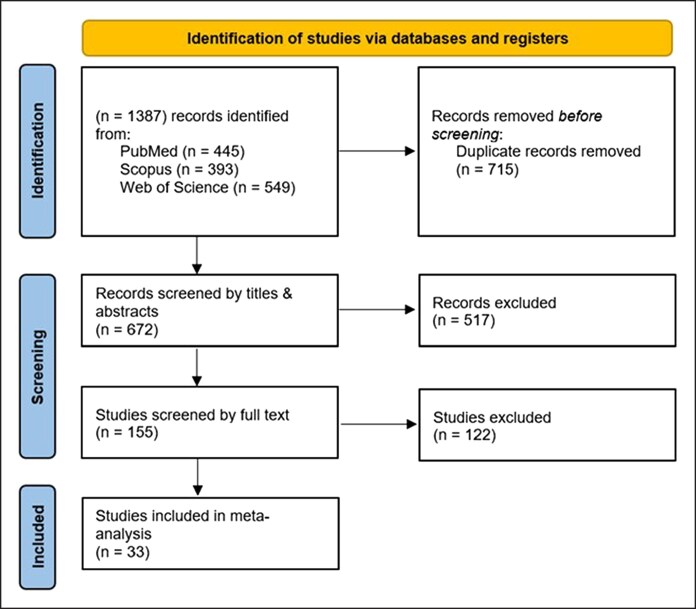
PRISMA flow diagram.

The comprehensive search (database inception to January 15, 2025) yielded 33 head-to-head comparative studies (Figure 1) that satisfied the eligibility criteria: out of these 33 studies, 20 studies were published in the year 2007 or later, and 13 were predated 2007.^5,8-10,16-31-44^ Together, these studies contributed data on 35,052 breast implants (16,002 smooth and 12,483 textured implants). Almost all were observational cohort series (prospective registries or retrospective chart reviews); only one small, randomized trial was identified. Cosmetic augmentation and postmastectomy reconstruction populations were both well represented, including direct-to-implant, 2-stage tissue-expander sequences, and mixed indications. Across the included comparative cohorts, the reported follow-up ranged from early (<12 months) to very long term (>5 years). Most studies provided ≥12-month surveillance, with many clustered between 12 and 24 months and a smaller subset reporting multi-year follow-up. Supplemental Table 1 summarizes all studies published in the year 2007 or later.

Regarding the risk of bias across included cohorts, the primary concern was confounding, particularly differences in implant pocket, adjuncts (eg, ADM), incision/site, and era/policy changes; several series also had nonblinded assessments of capsular contracture. Objective endpoints (infection requiring treatment and implant loss/rupture) were generally at lower risk. These limitations motivated our plane-matched subgroup analyses and era-restricted sensitivity analyses.

### Outcomes

#### Capsular Contracture

**Figure 2.**
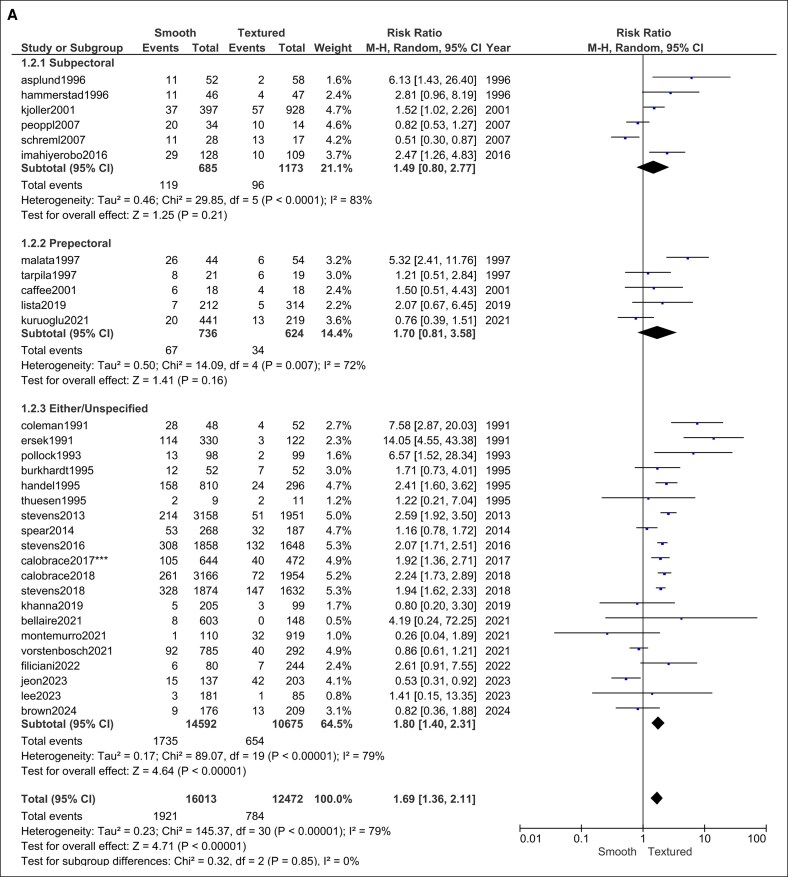

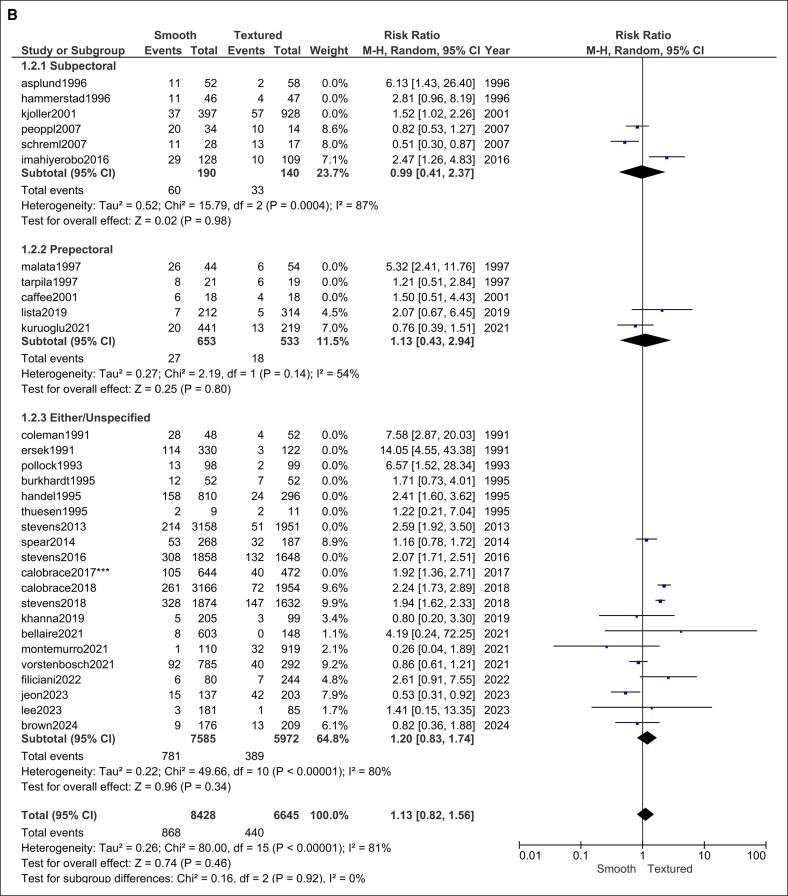
Capsular contracture forest plot (A) prior to and (B) after sensitivity analysis.

The overall pooled analysis of 31 comparative studies reporting capsular contracture (16,002 smooth vs 12,483 textured implants) demonstrated a significantly higher rate in the smooth group (RR = 1.69, 95% CI, 1.36-2.11, *P* < .00001). Heterogeneity was substantial (*I*^2^ = 79%; χ^2^ = 145.37, *P* < .00001; Figure 2A).

A subgroup analysis stratifying based on plane of implant placement revealed that the higher risk of capsular contracture was not statistically significant in both subpectoral (RR = 1.49, 95% CI, 0.80-2.77, *P* = .21; *I*^2^ = 83%; χ^2^ = 29.85, *P* < .0001; Figure 2A) and prepectoral planes (RR = 1.70, 95% CI, 0.81-3.58, *P* = .16; *I*^2^ = 72%; χ^2^ = 14.09, *P* = .007; Figure 2A). Regarding the prepectoral plane subgroup, heterogeneity was completely eliminated after sensitivity analysis excluding Malata et al (*I*^2^ = 0%, *P* = .45) while still maintaining a statistically insignificant risk of contracture (RR = 1.12, 95% CI, 0.72-1.74, *P* = .61).^41^

Further, another sensitivity analysis led to the exclusion of all studies published prior to the year 2007, as well as the studies by Stevens et al and Calobrace et al.^9,10,20^ The latter 3 were excluded because of overlapping with more recent cohorts (Stevens),^8^ inadequate stratification when reporting capsular contracture, and lack of direct implant texture comparisons between matched cohorts causing significant heterogeneity and potential confounding.^19^ The subsequent analysis revealed no statistically significant difference in the risk of capsular contracture between smooth and textured breast implants for each subgroup (subpectoral, prepectoral, and either/unspecified) as well as in the combined sample of all subgroups (RR = 1.13, 95% CI, 0.82-1.56, *P* = .46) with no subgroup differences (*I*^2^ = 0%, *P* = .92). Combined heterogeneity remained significant (*I*^2^ = 81%, *P* < .00001) as expected (Figure 2B).

The trend toward fewer contractures with time in smooth implant cohorts may reflect the higher prevalence of ADM use, particularly in the prepectoral plane; nonetheless, ADM utilization was inconsistently reported and therefore could not be meta-regressed.

#### Seroma

**Figure 3.**
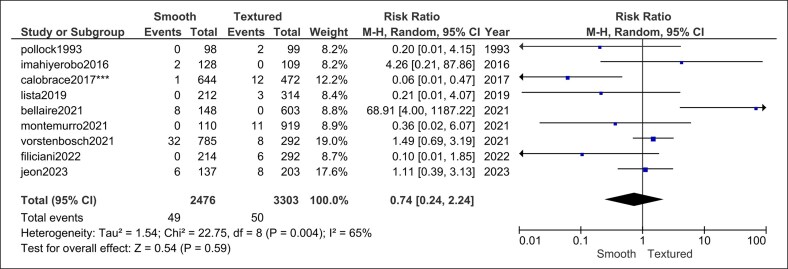
Seroma forest plot.

Pooling 9 trials (2476 smooth vs 3303 textured implants) showed no significant difference in seroma formation (RR = 0.74, 95% CI, 0.24-2.24, *P* = .59). Heterogeneity was significant (*I*^2^ = 65%, *P* = .004; Figure 3).

#### Hematoma

**Figure 4.**
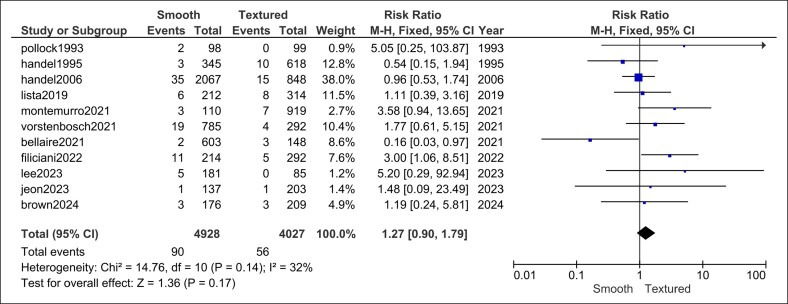
Hematoma forest plot.

Across 11 studies (4928 smooth vs 4027 textured implants), hematoma risk did not differ (RR = 1.27, 95% CI, 0.90-1.79, *P* = .17). Heterogeneity was low (*I*^2^ = 32%, *P* = .14; Figure 4).

#### Infection

**Figure 5.**
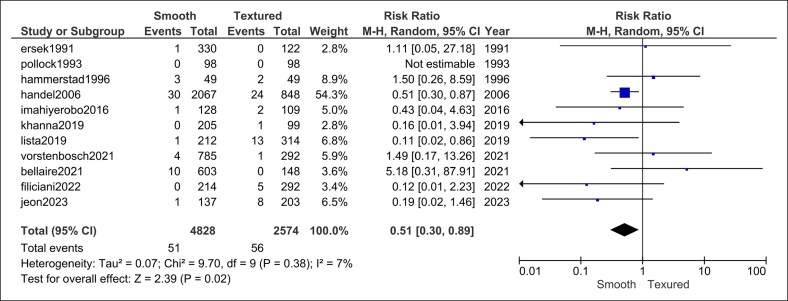
Infection forest plot.

Ten comparative cohorts (4828 smooth vs 2574 textured implants) reported postoperative infection. The pooled analysis favored smooth implants (RR = 0.51, 95% CI, 0.30-0.89, *P* = .02) with low heterogeneity (*I*^2^ = 7%, *P* = .29; Figure 5).

#### Asymmetry

**Figure 6.**
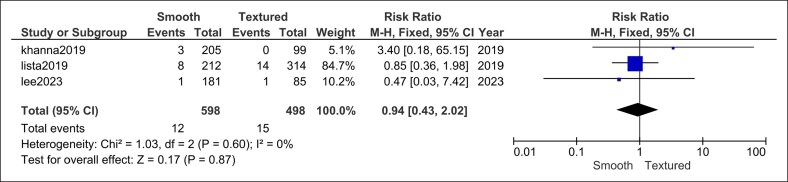
Asymmetry forest plot.

Three modern series (598 smooth vs 498 textured implants) demonstrated equivalent risks of postoperative asymmetry with no heterogeneity (RR = 0.94, 95% CI, 0.43-2.02, *P* = .87; *I*^2^ = 0%, *P* = .60; Figure 6).

#### Explantation

**Figure 7.**
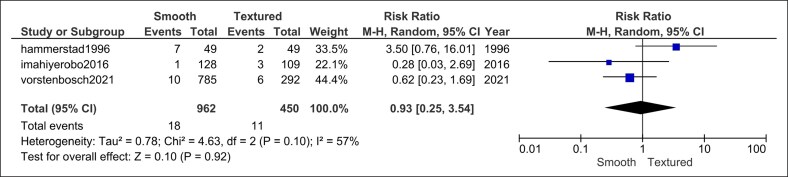
Explantation or implant loss forest plot.

Three studies (962 smooth vs 450 textured implants) revealed no significant difference in explantation rates with low heterogeneity (RR = 0.93, 95% CI, 0.25-3.54, *P* = .92; *I*^2^ = 57%, *P* = .10; Figure 7).

#### Device-Specific Events

**Figure 8.**
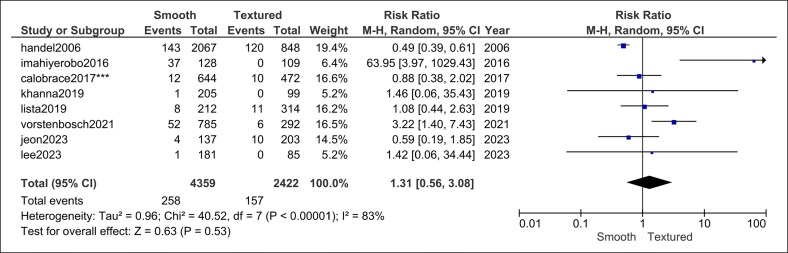
Implant rippling forest plot.

Rippling: 8 studies (4359 smooth vs 2422 textured implants) produced a neutral estimate (RR = 1.31, 95% CI, 0.56-3.08, *P* = .53) and significant heterogeneity (*I*^2^ = 83%, *P* < .00001; Figure 8). Heterogeneity could not be resolved by sensitivity analysis.

**Figure 9.**
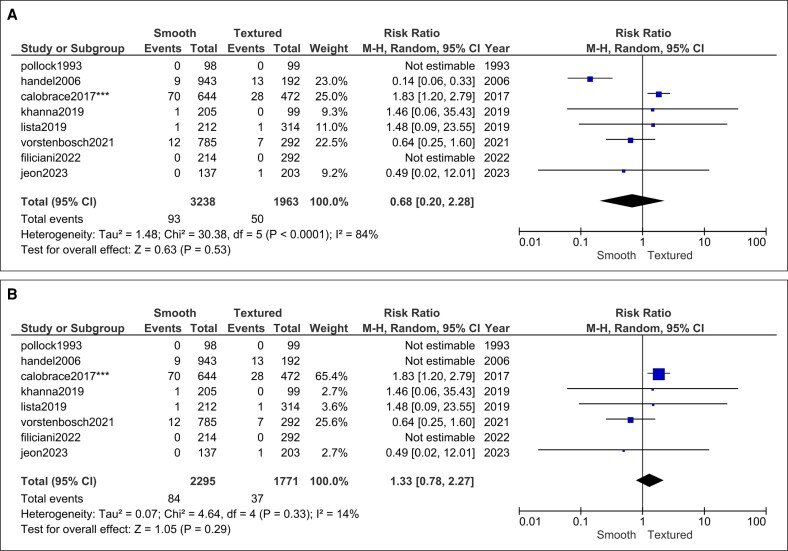
Implant rupture forest plot (A) before and (B) after sensitivity analysis.

Rupture: 8 studies (3238 smooth vs 1963 textured implants) produced a neutral estimate (RR = 0.68, 95% CI, 0.20-2.28, *P* = .53) and significant heterogeneity (*I*^2^ = 84%, *P* < .0001; Figure 9A). Sensitivity analysis led to the exclusion of Handel et al which resolved the heterogeneity (*I*^2^ = 14%, *P* = .33) and maintained a statistically insignificant risk (RR = 1.33, 95% CI, 0.78-2.27, *P* = .29; Figure 9B).^39^

**Figure 10.**
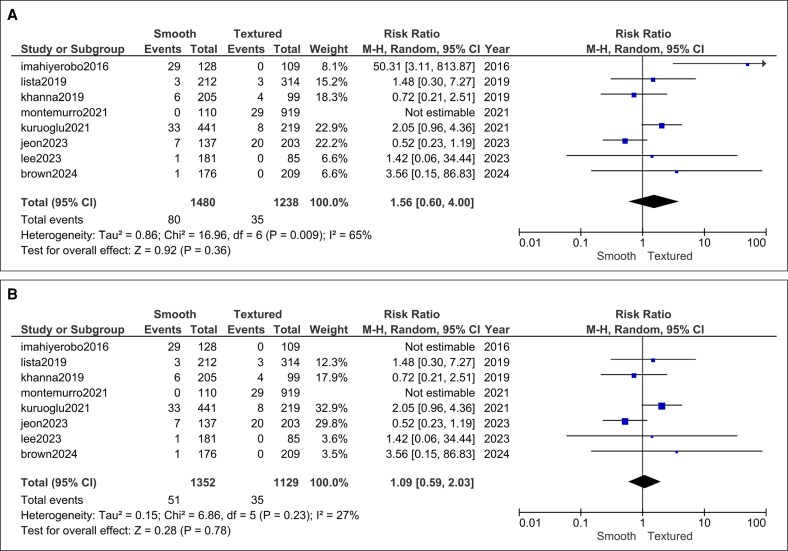
Implant malposition forest plot (A) prior to and (B) after sensitivity analysis.

Malposition/rotation: 8 recent cohorts (1352 smooth vs 1129 textured implants; Montemurro et al was excluded because the malposition was not reported for smooth implants) reported comparable malposition rates (RR = 1.56, 95% CI, 0.60-4.00, *P* = .36) with significant heterogeneity (*I*^2^ = 65%, *P* = .009; Figure 10A).^27^ Subsequent sensitivity analysis resolved the heterogeneity by excluding Imahiyerobo^21^ (*I*^2^ = 27%, *P* = .23) and the risk of malposition remained statistically insignificant (RR = 1.09, 95% CI, 0.59-2.03, *P* = .78).

#### BREAST-Q Outcomes

**Figure 11.**
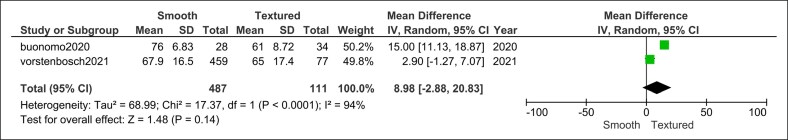
BREAST-Q breast satisfaction forest plot.

**Figure 12.**
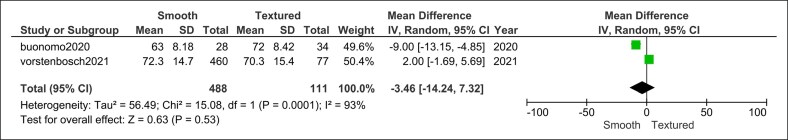
BREAST-Q physical well-being forest plot.

**Figure 13.**
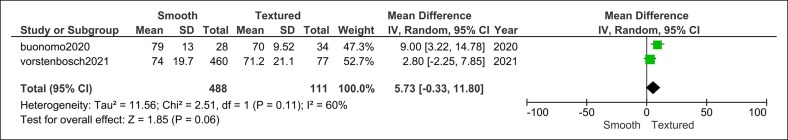
BREAST-Q psychosocial well-being forest plot.

Only 2 high-quality studies contributed patient-reported scores.^18,31^ None of the differences reached statistical significance (Table 1, Figures 11-13).

**Table 1.** BREAST-Q Outcomes

Domain	Mean difference	95% CI	*P*-value	*I* ^2^ (%)
Breast satisfaction	+8.98	−2.88 to 20.83	.44	94
Physical well-being	−3.46	−14.24 to 7.32	.63	93
Psychosocial well-being	+5.73	−0.33 to 11.80	.06	60

## DISCUSSION

Our findings suggest that smooth implants do not incur a higher risk of capsular contracture and appear to reduce postoperative infection compared with textured implants. Paired with similar results in other outcome areas, these findings would support smooth implants as a safer option for both aesthetic and reconstructive breast surgery.

Initially, analyses considering capsular contracture as an outcome found rates statistically higher in the smooth implant group compared with textured. Historical thinking mirrors this initial finding, whereby the smooth surface was believed to promote the formation of a concentric capsule and allow more implant mobility within the surrounding pocket allowing capsular compression and shear leading to fibrous tightening. Previous meta-analyses have also suggested that textured implants may confer a reduced association with capsular contracture.^5-7,45^ Heterogeneity was high, and upon further investigation with sensitivity analyses, we found that 3 studies were contributing to a great proportion of the increased heterogeneity. Two of those appeared to be using overlapping cohorts of patients and showed inadequate stratification when reporting capsular contracture and lack of direct implant texture comparisons between matched cohorts. After stratifying by implant pocket (submuscular vs subglandular/prepectoral) that been shown in other studies to impact the rate of capsular contraction, and excluding historical or overlapping cohorts, no statistically significant difference in capsular contracture risk was observed.^4,6^ Thus, our data refine the conventional wisdom by suggesting that any apparent benefit of texture on contracture is confounded by other variables (eg, pocket location) and is not seen in well-matched comparative analyses. This is consistent with other studies that suggest there is no difference in capsular contraction rates between textured vs smooth implants.^11,17,43^

Notably, smooth implants were associated with a significantly lower risk of postoperative infection (RR = 0.51, 95% CI, 0.30-0.89, *P* = .02) compared with textured implants. Theoretically, this is secondary to reduced surface area and lower bacterial adherence and biofilm formation.^46^ Some other studies show no significant difference between the two.^16,47,48^ Nelson et al reported an increased rate of infection with smooth capsules.^49^ There are, of course, multiple factors associated with infection that are often not reported and are not accounted for in this analysis, including patient demographics, ADM use, immediate or delayed reconstruction, antibiotic pocket irrigation, and drain use. However, in our analysis, heterogeneity was low (*I*^2^ = 7%, *P* = .29; Figure 5), suggesting the studies have a low degree of variability.

All other common complications and patient-reported outcomes were statistically equivalent between groups. For instance, no differences emerged in seroma, hematoma, implant malposition, rupture, or any BREAST-Q satisfaction domains. The latter is consistent with the existing evidence base.^31^

A pivotal context for these findings is the well-documented concern about BIA-ALCL. Virtually, all cases of BIA-ALCL have been linked to textured implants.^50^ In practical terms, this means that avoiding textured surfaces virtually eliminates the risk of BIA-ALCL. Increasing patient anxiety over ALCL means surgeons have incorporated implant surface into risk–benefit discussions, and those discussions tend to strongly favor smooth implants when balancing safety profiles.

Most cohorts in this review reported at least 12 months of follow-up, which is appropriate for detecting clinically meaningful capsular contracture and early implant-related events. Nonetheless, heterogeneity in surveillance windows persisted (12-24 months vs ≥2-5 years in others), and a minority of series reported <12 months. Because event risks accumulate with time, shorter follow-up can underestimate absolute rates and attenuate between-surface differences, whereas unequal follow-up between comparison arms within individual studies can bias estimates toward the longer-observed group. Our plane-matched and era-restricted sensitivity analyses reduced (but could not eliminate) these time-at-risk imbalances, so pooled estimates should be interpreted with this constraint in mind.

### Limitations

This meta-analysis is limited primarily by the retrospective and heterogeneous nature of most included studies. Across cohorts, there was substantial variation in study design, indications (augmentation vs reconstruction), implant plane (subpectoral, prepectoral, and mixed), adjuncts (eg, ADM), surgical technique, device generation/manufacturer, and follow-up duration.^51^ These differences likely contributed to the high between-study heterogeneity observed for several outcomes (eg, capsular contracture) and introduced residual confounding that cannot be fully eliminated by subgrouping or sensitivity analyses. Further, the majority of studies had either mixed or unspecified planes of implant placement as well as indications (augmentation vs reconstruction), rendering it difficult to stratify or conduct adequate subgroup analyses. Pocket definitions were inconsistently separated into subglandular vs subfascial in augmentation; most reports pooled these planes, precluding subset analysis. Incision/insertion site (eg, inframammary fold vs periareolar) was rarely reported and could not be analyzed. Outcome definitions were not uniform (eg, malposition and infections), raising the possibility of misclassification bias. Reporting of potentially influential covariates (eg, ADM use, pocket irrigation/antibiotics, drain management, immediate vs delayed reconstruction) was inconsistent, precluding meta-regression and limiting adjustment for center- or surgeon-level effects. Although we performed predefined sensitivity analyses (eg, excluding overlapping or historical registry cohorts and stratifying by implant plane) to reduce bias and improve clinical comparability, these methods mitigate but do not abolish confounding inherent to observational data. Patient-reported outcomes were informed by a few contemporary, high-quality studies, limiting precision for BREAST-Q estimates. Further, cosmetic results could not be assessed or meta-analyzed because of inadequate reporting and heterogeneity. Finally, rare events (eg, BIA-ALCL and rupture in modern devices) were not powered for definitive comparative risk estimates within this framework. Consequently, causal inference should be made cautiously, and our findings should be interpreted as the best available synthesis of head-to-head evidence rather than the equivalent of randomized data. Future work would benefit from large, prospective comparative cohorts or randomized trials using standardized definitions, protocolized perioperative care, device-level detail, and longer follow-up.

## CONCLUSIONS

After accounting for confounding factors, smooth implants do not increase capsular contracture risk, especially when placed submuscular, and appear to have reduced association with infection compared with textured implants. When these findings are combined, the risk–benefit profile of smooth implants supports their use over textured implants in aesthetic and reconstructive breast surgery. Nonetheless, given the predominance of retrospective data and incomplete confounder reporting, large comparative propensity-matched cohorts are needed to account for other confounders.

## Supplemental Material

This article contains supplemental material located online at https://doi.org/10.1093/asjof/ojag032.

## Supplementary Material

ojag032_Supplementary_Data
