# Supplementary material for: A Head-to-Head Meta-Analysis of 35,052 Smooth vs Textured Implants in Breast Reconstruction and Augmentation
Source: Aesthet Surg J Open Forum. 2026 Feb 12;8:ojag032. doi: 10.1093/asjof/ojag032 (PMC12997531; doi:10.1093/asjof/ojag032)
Supplement: ojag032_Supplementary_Data [file ojag032_supplementary_data.zip › Supplemental_Table_1.docx]

| Study (year) – population & intervention | N implants (smooth / textured) | Mean or median follow-up | Baker III–IV capsular-contracture smooth | Baker III–IV capsular-contracture textured |
| --- | --- | --- | --- | --- |
| Bellaire 2021 – direct-to-implant reconstruction | 603 / 148 | 118 w vs 96 w (mean) | 1.3 % (8 / 603) | 0 % (0 / 148) |
| Brown 2024 – sub-fascial cosmetic augmentation | 176 / 209 | 164 w vs 186 w (mean) | 5.1 % (9 / 176) | 6.2 % (13 / 209) |
| Buonomo 2020 – 1-yr reconstr. cohort (Italy) | 214 / 292 | 12 mo | NR | NR |
| Filiciani 2022 – primary augmentation (Argentina) | 214 / 292 | 12 mo | 4.7 % (10 / 214) | 1.4 % (4 / 292) |
| Imahiyerobo 2016 – nipple-sparing reconstr. | 128 / 109 | 24 mo | 23 % | 9 % |
| Jeon 2023 – DTI post-mastectomy reconstr. (Korea) | 137 / 203 | 29 mo | 10.9 % | 20.7 % |
| Kuruoglu 2021 – pre-pectoral reconstruction | 441 / 219 | 22 mo | 4.5 % | 5.9 % |
| Calobrace 2018 – 10-yr Sientra core study | 3184 / 1938 | 10 y | 8.2 % | 3.7 % |
| Khanna 2019 – Canadian Inspira augment. | 205 / 99 | 3 y | – (capsule 33 % of re-ops) | – |
| Lee 2023 | 181 / 85 | 11 mo vs 13 mo (mean) | 1.7% | 2.4% |
| Lista 2019 | 423 / 628 | 2 y vs 1.26 y (mean) | 3.2% | 1.59% |
| Montemurro 2019 | 220 / 1838 | 15 mo | 0.9% | 3.5% |
| Stevens 2018 | 1873 / 2230 | 10 y | 17.5% | 9% |
| Poeppl 2007  –  sub-muscular augmentation using Mentor gel implants; prospective comparative pathology study | 34 / 14 | 2 mo – 20 y (median not stated) | — | — |
| Spear 2014 (Natrelle Core, 10-y)  –  455 primary augmentation pts, Allergan Natrelle round gel; smooth vs BIOCELL textured | not given (≈ 59 % smooth, 41 % textured) | 10 y (Kaplan–Meier) | 19.9 % | 17.2 % |
| Stevens 2013 (Sientra, 5-y)  –  2560 primary augmentation pts, round gel implants; risk-factor study | 3158 / 1951 | 5 y (Kaplan–Meier) | 6.8 % | 2.6 % |
| Stevens 2016 (Sientra, 9-y)  –  1788 pts (all indications), round ± shaped gel implants; core study | 1865 / 1641 | 9 y (Kaplan–Meier) | 16.6 % | 8.0 % |
| Vorstenbosch 2021  –  1077 immediate reconstructions (TE → implant) at MSK; smooth vs textured | 785 / 292 | Median follow-up 1710 d (smooth) vs 2945 d (textured) | 11.7 % (92/785) | 13.7 % (40/292) |
| Coleman 1991 | 45 / 52 | 12 months | 58.3% (28/48) | 7.7% (4/52) |
| Ersek 1991 – Aesthetic augmentations  – Majority subglandular; some retromuscular subgroup analysis. | 330 / 122 | Smooth: 36 mo \| Textured: 12 mo | 34.5% (114/330) | 2.5% (3/122) |
| Pollock 1993  – Cosmetic bilateral augmentations only. – Smooth low-bleed double-lumen vs SILTEX textured silicone implants; all subglandular; same surgeon, same era. | 98 / 99 | Smooth: 21.5 mo \| Textured: 16.5 mo | 13.3% (13/98) | 4% (4/99) |
| Burkhardt 1995  – cosmetic re-augmentation, saline, subglandular | 52 / 52 | 20 mo | 23.1% (12/52) | 13.5% (7/52) |
| Handel 1995  – Very large single-center series of mixed indications (augmentation + reconstruction) and multiple implant types (smooth, textured, polyurethane, various fills and positions) | 810 / 296 | 27.6 mo | 19.5% (158/810) | 8.1% (24/296) |
| Thuesen 1995  – 20 unilateral post-mastectomy reconstructions, all subpectoral. – Randomized to smooth vs textured expander + implant | 9 / 11 Patients | NA | 22.2% (2/9) | 18.2% (2/11) |
| Asplund 1996 | 52 / 58 | 12 months | 21.2% (11/52) | 3.4% (2/58) |
| Hammerstad 1996 | 49 / 49 | Mean 32 months (textured) vs 44 months (smooth) | 23.9% (11/46) | 8.5% (4/47) |
| Tarpila 1997 | 21 / 21 | 6 and 12 months | 38.1% (8/21) | 28.6% (6/21) |
| Malata 1997 | 44 / 54 | 3 y | 59.1% (13/21 patients) | 14.3% (3/22 patients) |
| Caffee 2001 | 18 / 18 | 7.5 y | 33.3% (6/18) | 22.2% (4/18) |
| Kjoller 2001 | 397 / 928 | 20 y | 9.3% (37/397) | 6.1% (57/928) |
| Handel 2006 | 2067 / 848 | 10 y | ≈ 35% (Kaplan-Meier at 10 y) | ≈ 35% (Kaplan-Meier at 10 y) |
| Shreml 2007 | 28 / 17 pateints | 20 y | 39.3% (11/28) | 76.5% (13/17) |

**Supplemental Table 1.** Summary of all included studies.
*OR>1 indicates higher odds with smooth vs textured unless otherwise specified.
CC = Capsular Contracture. NS = Not Significant. OR = Odds Ratio. Mo = Months. Y = Years. W = weeks*
